# Can the Use of Bedside Lung Ultrasound Reduce Transmission Rates in The Case of The COVID-19 Patient? - A Narrative Review

**DOI:** 10.24908/pocus.v5i1.14227

**Published:** 2020-07-16

**Authors:** Sheena Bhimji-Hewitt

**Affiliations:** 1 The Michener Institute of Education at UHN Toronto, Ontario Canada

**Keywords:** POCUS, COVID-19

## Abstract

Novel Corona Virus Disease-19 (nCov-19, COVID-19) was recognised as a pandemic by the World Health Organization on March 11, 2020. As of June 14, 2020, this contagious viral disease has afflicted 188 out of 195 countries in the world with 7,893,700 confirmed cases and 432,922 global deaths.Canada has 98,787 people infected and 8,146 deaths. COVID-19 is thought to transmit through contact, droplets and aerosolization. A rapid review showed limited information on the benefits of conducting lung ultrasound (LUS) versus chest radiograph (CXR) or studies correlating lung ultrasound to chest computed Tomography (CT) in patients positive for Covid-19. The literature review confirmed that CT and LUS cannot diagnose this disease, but that both can help in the management and staging of this disease. There is no literature to prove that LUS at the bedside may be beneficial from the view of decreased transmission to other health care workers and bystanders due to reduced transit but comparing the transit pathway and contact leads one to propose that this would be so. Pregnant patients with COVID-19, young children and patients in the reproductive stage would also benefit from LUS since there is no radiation dose and the critical patient in distress will benefit from testing at the bedside.

## Introduction

On March 11, 2020, the World Health Organization (WHO) declared Corona Virus Disease 2019 (COVID-19) a pandemic 1. This declaration and the virus has impacted the world; as of June 14, 2020; 188 out of 195 countries have been impacted, there are 7,893,700 confirmed cases, an increase of over 5 million since April 18, 2020, and 432,922 global deaths (156,064 April 18, 2020) 2. Canada reported 98,787 confirmed infections on June 14, 2020; this number has tripled in 2 months and 8,146 deaths, a six-fold increase from April 18, 2020 3.

COVID-19 is predominantly a respiratory disease, with severity ranging from mild to fatal 4. Transmission is thought to be by contact, droplets and aerosol generating medical procedures (AGMP) 3, 5. COVID-19 findings are consistent with a viral pneumonia caused by viral infections such as Severe Acute Respiratory Syndrome (SARS) or Middle East Respiratory Syndrome 4, 6, 7.

## Methods

Articles were extracted from PubMed, Ovid, and Google Scholar, to identify meta-analysis, original research and case reports. Information from government and educational websites such as The World Health Organization, Canadian provincial government resources and guidelines from the American college of Radiology (CAR) were also reviewed for current statistics and recommendations.

The last search of each database was conducted on June 14, 2020.

The key search terms were lung ultrasound, COVID-19, Corona virus, personal protective equipment, lung ultrasound pregnancy, bedside ultrasound, using the Boolean operator AND. Medical Subject Heading (MeSH) terms (lung ultrasound AND ultrasonography AND bedside ultrasound AND covid-19, AND Corona Virus AND or Computer Tomography AND or CTs) were used in both PubMed and OVID. Limits used in both PubMed and MEDLINE were to include English articles. In all databases, limits were set to publication dates within the last 5 years (2015-2020). The evidence that was retrieved was quite weak due to the very limited availability of rigorous original research with most evidence coming from case studies and gray literature.

## Discussion

Reports out of China and Italy, two of the first and hardest hit countries in the world (on April 18, 2020) reported positive results for the use of lung ultrasound to confirm lung consolidation 4, 8, 9 . To date LUS has been utilized to diagnose for respiratory conditions such as pneumonia, pleural effusions and pneumothorax and been utilized at the patient’s bedside 10, 11. Convissar et al’s narrative review compared the findings from 2 original research studies and 3 case reports of patients that had tested positive for COVID-19; their conclusion was “ultrasonography has repeatedly proven to be an effective imaging modality to aid in both diagnosing and monitoring the progression of viral pneumonias” 11. Lung ultrasound cannot be utilized to distinguish COVID-19 from any other viral infection but it can “differentiate between a possible pneumonia and alternative causes of dyspnea while awaiting confirmatory testing or in areas where molecular assays are unavailable” 11. Serial ultrasounds can also aid in tracking the clinical path and guide appropriate treatment or interventions 11. 

Computed Tomography (CT) of the chest is the best diagnostic imaging for lung consolidation; however, it is not recommended for diagnostic use in COVID-19 12, 13. Instead it is recommended to be used as a diagnostic test in cases where it will impact patient management, or to evaluate for urgent or emergent conditions 14, 7. Use of this diagnostic test requires transfer of patients to and from the CT scanner, potentially exposing transportation staff, staff in the CT room as well as passersby 14, 11, 12. Patients having this test are exposed to radiation and it is contraindicated for pregnant patients 12, 13. Proper disinfection of this unit can be lengthy; limiting access to the equipment, as many hospitals have only 1 or 2 CT suites 7, 12. Chest radiographs (CXR) may be used but have the same transit path if not portable, and regardless of portable or radiology suite based, it will require the same disinfection and contraindications due to the radiation dose 4, 12. The American College of Radiology (ACR) recommends that Chest CT not be used to screen for or diagnose COVID-19 and that it be used sparingly in hospitalized or symptomatic patients to decrease the risk of infection transmission 12, 15.

**Figure 1:**
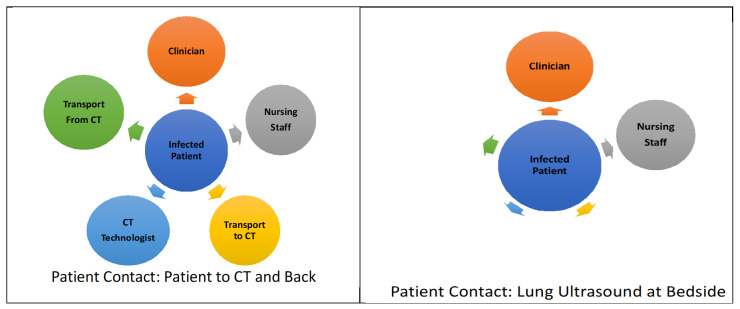
Comparison of Patient contact

Lung ultrasound (LUS) if performed at the patient’s bedside (Figure 1) will reduce patient transit, contact with additional health care workers (HCWs) and medical devices 4, 6, 9, 11. This in turn may decrease human resources and personal protective equipment used, thus providing more efficient, higher value care for the COVID-19 patient 11. Ultrasound performed at the patient’s bedside is also more feasible for the critical patient that cannot be moved to the CT scanner. LUS is without radiation exposure which is of extra benefit in pregnant patients, young children and patients in the reproductive age where CT radiation is contraindicated or should be limited. Disinfection of the portable or hand-held ultrasound devise is easier and more efficient to complete than that for a CT or CXR suite 4, 12. Finally, lung ultrasound at the bedside may provide more compassionate care to the patient who is in physical or emotional distress.

## Conclusions

COVID-19 is an unprecedented, extremely contagious disease in modern times. Chest CT has been historically utilized for lower respiratory tract infections and has excellent diagnostic value, but at this time ACR recommendations are to limit its use. LUS can be utilized for imaging and assessing for lung consolidation; however, there is little research or reports to establish the correlation between the 2 imaging tests or the sensitivity or specificity for lung ultrasound in COVID-19 patients. Regardless, LUS could potentially reduce exposure and thus transmission to HCWs, reduce human resources and provide more efficient care. It is also ideal for patients in whom CT is not recommended, or would not tolerate transfer to the CT suite. 

For some, LUS is a new diagnostic imaging test emerging in this pandemic; for others it has been used for years. This diagnostic imaging test may prove to be valuable but more rigorous research and reporting needs to be done. 

## Conflicts of Interest

None declared.
